# Haptoglobin Is Required to Prevent Oxidative Stress and Muscle Atrophy

**DOI:** 10.1371/journal.pone.0100745

**Published:** 2014-06-24

**Authors:** Enrico Bertaggia, Gaia Scabia, Stefania Dalise, Francesca Lo Verso, Ferruccio Santini, Paolo Vitti, Carmelo Chisari, Marco Sandri, Margherita Maffei

**Affiliations:** 1 Department of Biomedical Sciences, University of Padova, Padova, Italy; 2 Dulbecco Telethon Institute at Venetian Institute of Molecular Medicine, Padova, Italy; 3 Department of Clinical and Experimental Medicine, University of Pisa, Pisa, Italy; 4 Unit of Neurorehabilitation, Department of Neuroscience, University Hospital of Pisa, Pisa, Italy; 5 CNR Institute of Neuroscience, Padova, Italy; 6 Telethon Institute of Genetics and Medicine (TIGEM), Napoli, Italy; 7 CNR Institute of Clinical Physiology, Pisa, Italy; University of Rome La Sapienza, Italy

## Abstract

**Background:**

Oxidative stress (OS) plays a major role on tissue function. Several catabolic or stress conditions exacerbate OS, inducing organ deterioration. Haptoglobin (Hp) is a circulating acute phase protein, produced by liver and adipose tissue, and has an important anti-oxidant function. Hp is induced in pro-oxidative conditions such as systemic inflammation or obesity. The role of systemic factors that modulate oxidative stress inside muscle cells is still poorly investigated.

**Results:**

We used Hp knockout mice (Hp^-/-^) to determine the role of this protein and therefore, of systemic OS in maintenance of muscle mass and function. Absence of Hp caused muscle atrophy and weakness due to activation of an atrophy program. When animals were stressed by acute exercise or by high fat diet (HFD), OS, muscle atrophy and force drop were exacerbated in Hp^-/-^. Depending from the stress condition, autophagy-lysosome and ubiquitin-proteasome systems were differently induced.

**Conclusions:**

Hp is required to prevent OS and the activation of pathways leading to muscle atrophy and weakness in normal condition and upon metabolic challenges.

## Background

Haptoglobin (Hp) is an acute-phase plasma glycoprotein produced mainly by hepatocytes [1] and adipocytes [2], and is the major hemoglobin binding serum protein in several mammals. The concentrations of Hp in the plasma are high, ranging from 0.3 mg/ml to 3 mg/ml, producing an Hp:Hb molar ratio of 400. By forming a complex with Hb, Hp prevents both iron loss and kidney damage during hemolysis. The Hp-Hb complex is transported to the liver and other tissues to be degraded by Hp-Hb scavenger receptors, such as the CD163 receptor present on macrophages and liver Kupfer cells [3]. An important aspect of Hb scavenging by Hp is the reduction in Oxidative Stress (OS). Indeed extracorpuscular Hb can initiate a free radical reaction by releasing heme iron, which acts as a potent Fenton reagent. This reaction results in the production of Reactive Oxygen Species (ROS) and consequent oxidative damage to tissues. Hp binding to Hb prevents this cascade by shielding the heme iron from its aqueous surrounding [3], [4]. A prominent aspect of mice lacking Hp is the oxidative damage suffered by their kidney in the presence of acute hemolysis provoked by phenylhydrazine injection. In the first comprehensive description of this mouse model Baumann and colleagues state that the most important physiological function of the strong Hb-Hp binding is to inhibit Hb-stimulated lipid peroxidation [5]. Indeed these mice show higher levels of the systemic OS indicators malonaldehyde (MDA) and 4-hydroxy- 2(E)-nonenal (HNE).

Hp is part of the acute phase response to inflammation for which is considered a marker in the clinical practice [6]. Hp is also specifically induced in the white adipose tissue upon obesity, this being reflected in the increased plasma levels of the glycoprotein found in obese subjects [2], [7]. The induction of Hp seen in chronic conditions typically associated to enhanced OS [8] might be a mechanism to prevent an excessive damage caused by free radicals. Noteworthy the two common alleles for Hp (1 and 2) present in humans possess different properties, with the protein encoded by Hp 1-1 providing superior antioxidant protection compared with that encoded by Hp 2-2. Diabetic individuals with Hp 2-2 have been reported to be more prone to develop nephropathy, retinopathy, and cardiovascular disease than those with the Hp 2-1 or Hp 1-1 genotypes [8].

If on one side Hp induction in conditions of enhanced OS, as in the adipose tissue of obese subjects, is protective, on the other its proven chemotactic potential for macrophages [9] augments the local inflammatory infiltrate. Indeed Hp absence diminishes local inflammation and enhances insulin sensitivity in the adipose tissue of obese (but not lean) mice and partially protects from obesity associated insulin resistance [10], [11]. Insulin response of skeletal muscle, that does not normally express Hp, was not affected by Hp deficiency [10]: a comprehensive characterization of this tissue has never been carried out and it is of interest to understand if Hp dosage impinges on skeletal muscle mass and function.

Oxidation can alter the structure and function of lipids, proteins and nucleic acids, leading to cellular injury and even cell death. Several lines of evidence suggest that OS leads to muscle atrophy, a feature often associated to conditions characterized by an impaired mitochondrial function [12]. Genetic evidence has shown that increased OS in skeletal muscle is sufficient to induce muscle atrophy [13], [14].

The muscle mass is affected by the balance between synthesis and degradation of intracellular components. Muscle atrophy occurs when the degradation rate is higher than the synthesis rate.

The Phosphatidylinositide 3-kinase (PI3K)/Akt signaling cascade plays a key role in the regulation of muscle mass [15], [16] and promotes fiber hypertrophy by stimulating protein synthesis and by suppressing proteolysis. In skeletal muscle, activation of Akt by Insulin-like Growth Factor (IGF1) stimulates protein translation through induction of Mammalian Target of Rapamycin (mTOR), which activates p70S6K and inactivates the inhibitor of translational initiation 4E-binding protein (4EBP1) [15], [16]. In addition, Akt suppresses muscle protein breakdown directly by phosphorylating the transcription factor FoxO3, leading to its inactivation through sequestration in the cytosol [17], [18]. During atrophy, the activity of the IGF-1/PI3K/Akt pathway decreases, permitting the translocation of FoxO3 into the nucleus, which leads to a stimulation of protein breakdown through the transcription of genes of the ubiquitin–proteasome pathway [17], [18] and of the autophagic/lysosomal system [19], [20]. These two proteolytic systems account for 90% of protein breakdown in mammals.

The ubiquitin-proteasome system (UPS) consists of concerted actions of enzymes that link chains of the polypeptide ubiquitin onto proteins to mark them for degradation in a large multicatalytic protease complex, the proteasome [21]. The rate limiting step in ubiquitination process is catalyzed by the class of E3 enzymes, known as ubiquitin ligases. Recently two muscle-specific atrophy-related ubiquitin ligases, Atrophy gene-1/Muscle Atrophy F-box (Atrogin-1/MAFbx) and Muscle Ring-Finger protein 1 (MuRF1), which are transcriptionally upregulated during early stages of muscle atrophy have been found [22].

The other proteolytic system that is activated in catabolic conditions and that is under FOXO regulation is macroautophagy, hereafter named autophagy [23]. Autophagy is a highly conserved homeostatic mechanism used for the degradation and recycling, through the lysosomal machinery of bulk cytoplasm, long-lived proteins and organelles [24]. The autophagy machinery generates double membrane vesicles (autophagosomes) that engulf and sequester target cellular components [25], [26]. Autophagosomes are then delivered to and fuse with lysosomes to degrade their contents. The autophagic process plays a crucial role in the turnover of cell components both in constitutive conditions and in response to various stimuli, such as cellular stress and nutrient deprivation. Expression of FoxO3 is sufficient and required to activate lysosomal-dependent protein breakdown in cell culture and *in vivo* [19]. Gain and loss of function experiments identified Bnip3, a BH3-only protein, as a central player downstream of FoxO [19], [27], [28].

In the present study we explored how lack of a factor that regulates systemic OS, like Hp, may impact on skeletal muscle size and function in both normal situation, and conditions that are known to increase ROS production and OS, either acutely, such as physical exercise, or chronically herein induced by High Fat Diet (HFD). The data revealed a critical role of Hp in preventing protein oxidation and weakness in skeletal muscle.

## Results

### Absence of Hp induces muscle atrophy and expression of atrophy-related genes

**Figure 1:**
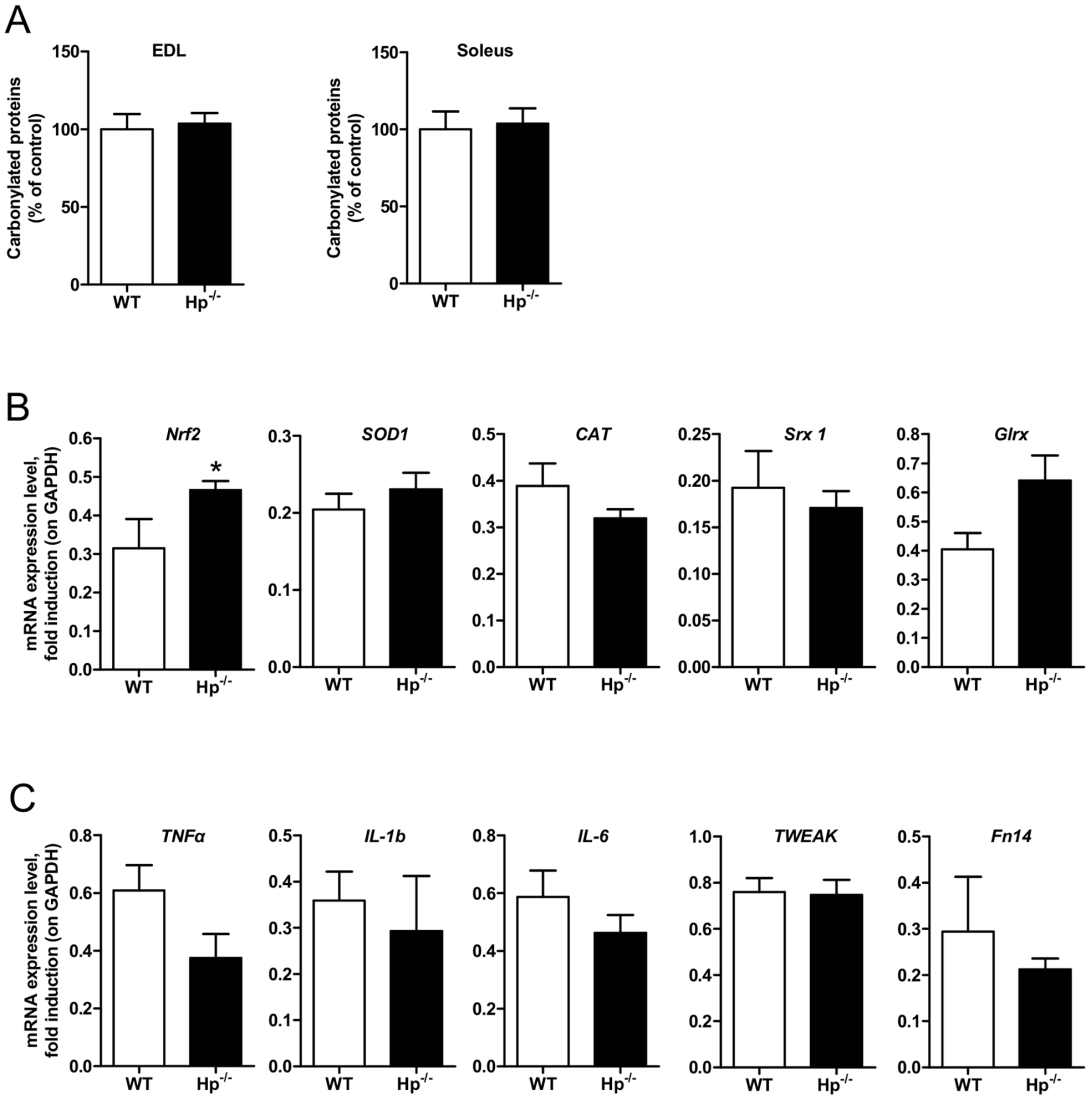
Haptoglobin deficiency effect on protein carbonylation, antioxidant response and inflammatory response in the skeletal muscle. A) Protein carbonylation is similar in *EDL* and *soleus* muscle of WT and Hp^-/-^ mice (n = 11). B) mRNA analysis for anti-oxidant response (names of genes as indicated) in *Tibialis anterior* muscle of WT and Hp^-/-^ mice (n = 8). C) mRNA analysis for inflammatory response (names of genes as indicated) in *Tibialis anterior* muscle of WT and Hp^-/-^ mice (n = 8). Data are expressed as mean ± SEM. Student's t-test: *P<0.05.

Previous studies from our lab showed that Hp deficiency does not result in any relevant metabolic abnormality under normal diet [10]. Indeed, the adipose tissue was similar between Hp^-/-^ and Wild Type (WT) mice in terms of mass and expression of inflammatory and mitochondrial biogenesis markers [10] (Maffei, unpublished work). Given the well established role of Hp as an antioxidant, and being OS an important regulator of muscle performance, we characterized skeletal muscle of Hp knockout mice. Initially, we monitored the level of OS by analyzing protein carbonylation in both fast-twitch (*Extensor Digitorum Longus, EDL*) and slow-twitch (*soleus*) muscle. In standard conditions Hp deficiency did not show any significant increase of oxidized proteins in any of the two muscles (Figure 1A, left and right panels). Interestingly, expression of *Nrf2*, an important transcription factor that is induced under OS to coordinate ROS detoxification [29], was higher in the skeletal muscle of Hp^-/-^ mice (Figure 1B). When we monitored the expression of some anti-oxidant genes, such as *Superoxide dismutase 1* (*SOD1*), *catalase* [30], *Sulfiredoxin* 1 (*Srx 1*), we did not find any significant induction. A trend of induction was observed in *Glutaredoxin* (*Glrx*), a key modulator of the intracellular redox state [31] (Figure 1B).

**Figure 2:**
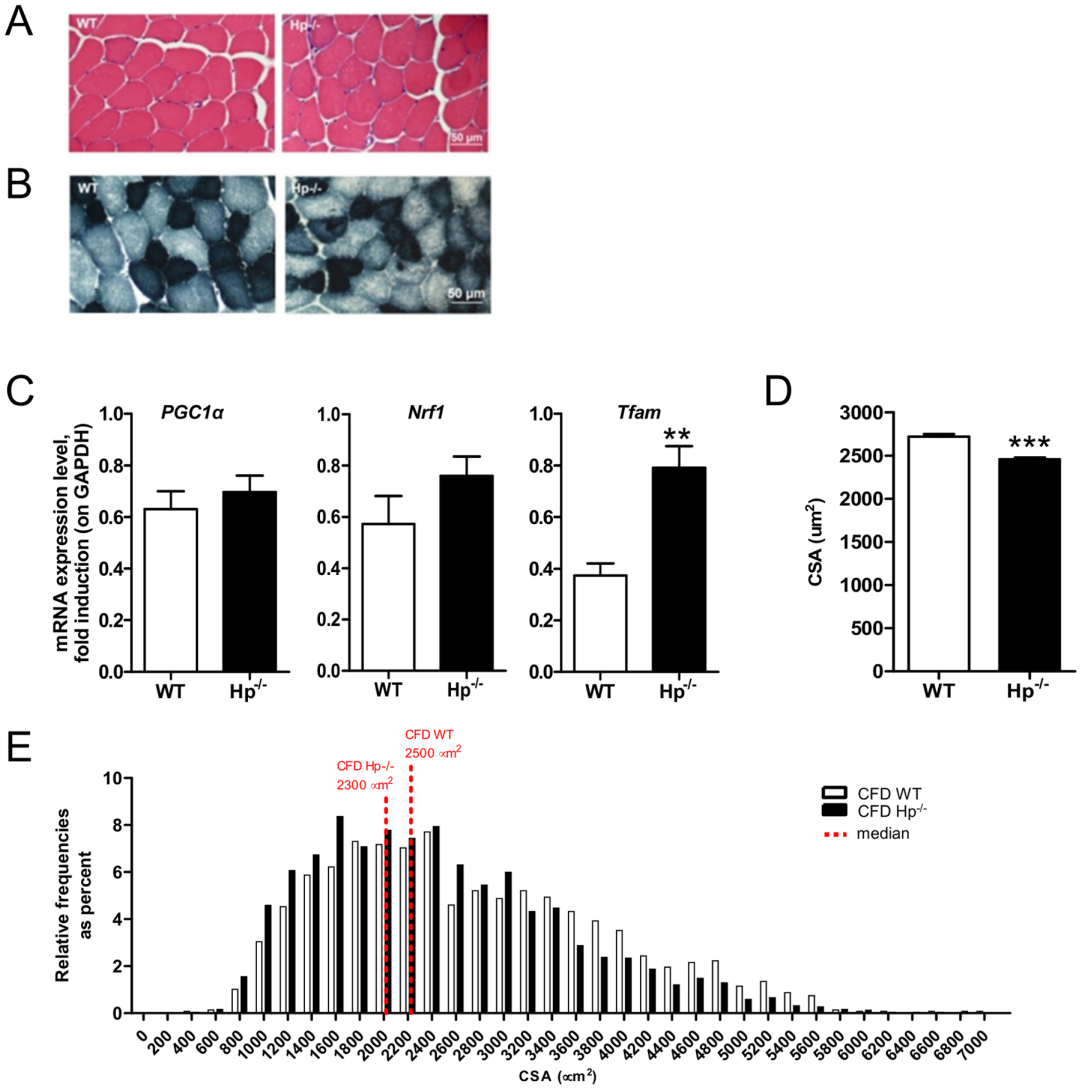
Haptoglobin deficiency induces muscle atrophy in skeletal muscle. A) Hematoxylin and Eosin staining of WT and Hp^-/-^ *Tibialis anterior* muscle shows no sign of fiber degeneration and/or inflammation. B) Succinate Dehydrogenase (SDH) staining of WT and Hp^-/-^ *Tibialis anterior* muscle shows no major differences of fiber types. C) mRNA expression levels of *PGC1α*, *Nrf1* and *Tfam* in *Tibialis anterior* of WT and Hp^-/-^ mice (n = 8). D) Cross sectional area (CSA) of *Tibialis anterior* is reduced in Hp^-/-^ (10%) as compared to WT mice (n≥2500 fibers). Muscles were dissected from 5 months old mice. E) Cross sectional area (CSA) frequency distribution of *Tibialis anterior* in Hp^-/-^ and WT mice. The bar graph represents relative frequencies as percent. Red-dotted lines indicates the median for each distribution (n≥2500 fibers). Data are expressed as mean ± SEM. Student's t-test: **P<0.01; ***P<0.001.

Since inflammation is a potent source of ROS [32], we checked the level of expression of different inflammatory cytokines in muscles of WT and Hp^-/-^ mice. No significant difference was found for *IL-6, IL-1b*, *Tumor Necrosis Factor α* (*TNF*α), *Tumor necrosis factor-like weak inducer of apoptosis* (*TWEAK*) and its receptor *FGF inducible 14 protein* (*Fn14*), suggesting that inflammation is not present in muscle of Hp knockout mice (Figure 1C). Morphological analysis confirmed the absence of inflammation and revealed no sign of fiber degeneration and regeneration (central nucleated fibers) in *Tibialis anterior* of Hp deficient mice (Figure 2A). SDH staining showed no major differences of fiber types. Indeed, the amount of small mitochondrial-rich beta-oxidative fibers (49.3% and 48.7% for WT and Hp^-/-^ respectively) and large mitochondrial-poor glycolytic fibers (50.7% and 51.3% for WT and Hp^-/-^ respectively) was identical between WT and Hp^-/-^ mice (Figure 2B). In line with SDH staining, *PGC1α* and *Nrf1* expression was not significantly different between WT and Hp^-/-^ animals (Figure 2C) while *Tfam* was induced in Hp^-/-^ mice (Figure 2C). When we monitored fiber size in animals of 5 months of age, we found a significant reduction of cross-sectional area (CSA) (10%) in *Tibialis anterior* of Hp^-/-^ mice when compared to age-matched controls, this effect being apparent both in the comparison between means (Figure 2D) and between CSA distributions (Figure 2E). Importantly, this reduction of myofiber size is due to atrophy since Hp-deficient myofibers of 3 months old animals were bigger than controls (WT = 2051±16.6, KO = 2575±18.8; P<0.001). This finding suggests that absence of Hp does not affect myogenesis and postnatal growth but it induces an atrophy program leading to myofibrillar protein loss in adulthood.

**Figure 3:**
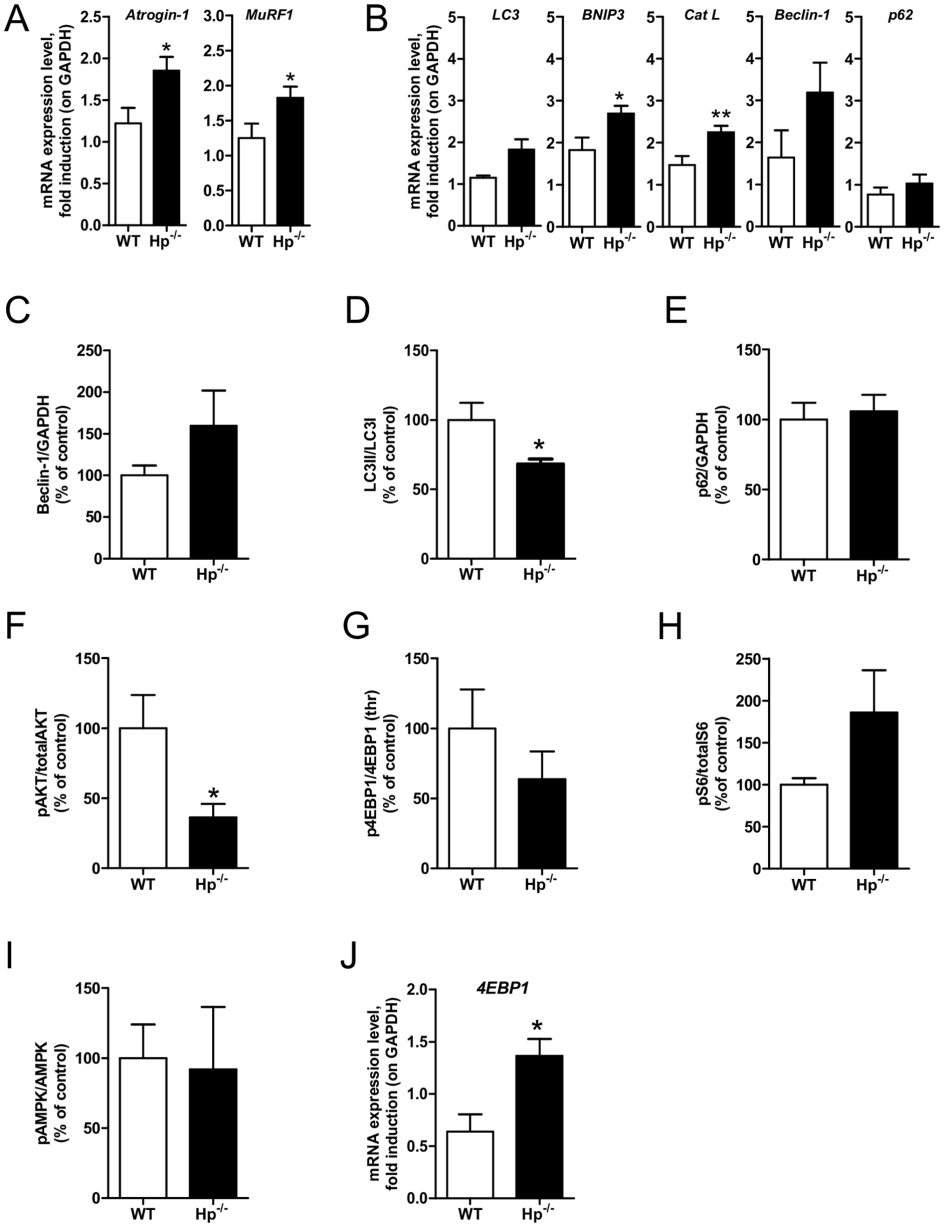
Haptoglobin deficiency induces activation of the ubiquitin-proteasome system and inhibition of protein synthesis in the skeletal muscle. A) *Atrogin-1* and *Murf1* mRNA transcripts are increased in Hp^-/-^ as compared to WT skeletal muscle. B) mRNA expression levels of autophagy related genes (*names of genes as indicated*) in the skeletal muscle of WT and Hp^-/-^ mice. (C-I) Western blots analysis on protein isolated from skeletal muscle of WT and Hp^-/-^ mice showing: C) Beclin-1 protein levels, D) LC3 lipidation, E) p62 protein levels, F) Akt phosphorylation (ratio between signal on Ser 473 and total Akt), G) 4EBP1 phosphorylation (ratio between signal on Thr 37/46 and total 4EBP1), H) S6 phosphorylation (ratio between signal on Ser 235/236 and total p70S6), I) AMPK phosphorylation (ratio between Thr172 and total AMPK). The bar graphs represent the quantification of the sum of experiments; data are expressed as percentage of “WT”, where WT is considered 100%. J) mRNA expression levels of *4EBP*1. Real time PCR and Western Blot analyses were respectively performed on *Tibialis anterior* cDNA (n = 8) and *Gastrocnemius* muscle lysates (n = 6). GAPDH is used as control of equal loading in both cases. Muscles were dissected from 5 months old mice. Data are expressed as mean ± SEM. Student's t-test: *P<0.05; **P<0.01.

The reduction in myofiber size might be a consequence of either inhibition of protein synthesis, activation of protein breakdown, or both. To reveal which mechanism is involved, we monitored the expression of atrophy-related genes belonging to the ubiquitin-proteasome and autophagy lysosome systems. *Atrogin-1* and *MuRF1*, the atrophy-related ubiquitin ligases, were significantly induced in Hp^-/-^ mice (Figure 3A). Similarly, when we monitored the expression of several autophagy related genes we found a significant upregulation of *Cathepsin L* (*Cat L*) and *Bnip3* (Figure 3B), while *LC3* and *Beclin-1* mRNAs showed a trend of induction. However, when we checked protein levels we observed a trend of increase of Beclin-1 protein, no changes in p62 protein and a slight decrease of LC3II/LC3I ratio (Figures 3C, 3D). Since p62 is the best *bona fide* substrate of autophagy and since the p62 mRNA was unchanged (Figure 3B), we can conclude that autophagy is not significantly induced in Hp null muscles (Figure 3E) compared to WT.

We then monitored the expression and activation of pathways related to protein synthesis. In line with the reduced size of muscle fiber, Akt phosphorylation was reduced in Hp^-/-^ (Figure 3F), while phosphorylation of mTOR downstream targets, such as 4EBP1 and S6, were not significantly changed (Figures 3G, 3H). Consistent with mTOR data, 5' AMP-activated protein kinase (AMPK) was not activated in Hp^-/-^ (Figure 3I). Interestingly, the absence of Hp induced expression of *4EBP1* transcript (Figure 3J) that may account for the slight decrease of the 4EBP1 phosphorylation (Figure 3G).

This first set of data suggests that the absence of Hp, even in absence of a significant OS, impinges on skeletal muscle mass by inducing an upregulation of atrophy-related genes, that are related to induction of ubiquitin-proteasome dependent protein breakdown. We next decided to study the effect of an acute boost of ROS production versus a chronic exposure to OS, represented respectively by exercise and obesity on the muscle phenotype of Hp^-/-^ mice.

### Endurance exercise promotes oxidative stress and weakness in Hp knockout mice, that do not show an adequate antioxidant response

**Figure 4:**
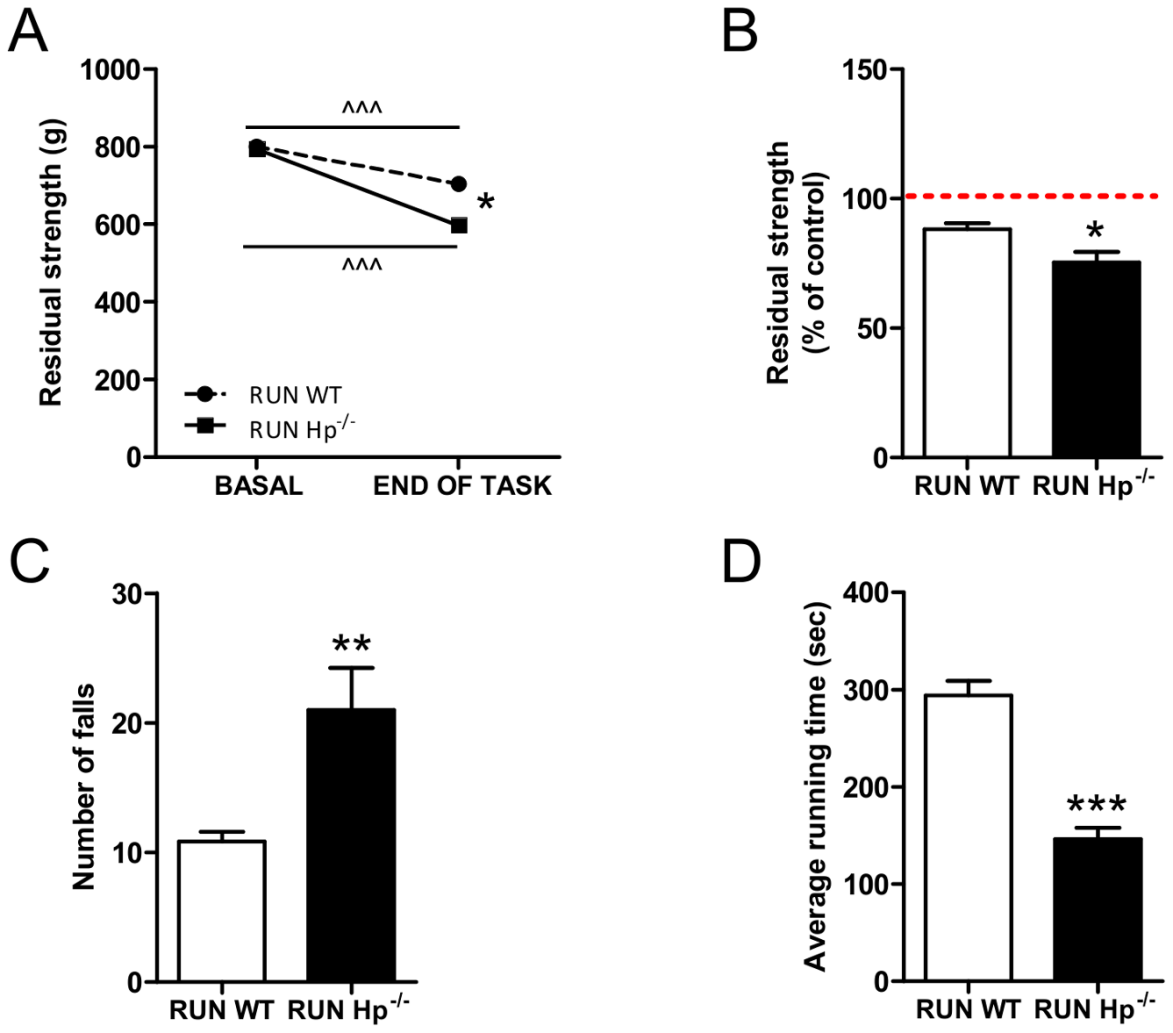
Haptoglobin deficiency affects muscle performance. A) and B) Forelimbs strength assessment by grip test. A) WT and Hp^-/-^ mice show overlapping basal strength; following rotarod exercise (end of task) Hp^-/-^ undergo a more pronounced strength reduction as compared to WT. B) residual strength, expressed as percentage of basal strength before the exercise (dashed line) is lower in Hp^-/-^ as compared to WT mice. C) Number of falls/hour from rotarod is higher in Hp^-/-^ mice (RUN Hp^-/-^) as compared to controls (RUN WT). D) The average consecutive time that mice spent on rotarod is significantly shorter in RUN Hp^-/-^ as compared to controls (RUN WT) (n = 8). Data are expressed as mean ± SEM. Student's t-test: *P<0.05; **P<0.01; ***P<0.001. Paired Student's t-test: ∧∧∧P<0.001.

To better understand the role of Hp, we challenged the animals with conditions that acutely promote ROS production in muscle such as endurance exercise. Muscle strength of WT and Hp^-/-^ mice was determined before and after the training. While untrained control and Hp^-/-^ showed no difference of force generation, the exercised Hp^-/-^ mice showed a significant drop of force when compared to WT animals (Figures 4A, 4B). Moreover, Hp^-/-^ mice fell more frequently than controls during each session (Figure 4C), and consequently the time that the animals spent on the rotarod between two consecutive falls was significantly shorter in Hp^-/-^ than in WT (Figure 4D). This finding confirms that Hp^-/-^ animals reach the fatigue before controls. Altogether these data demonstrate that the absence of Hp determines a decrease in muscle force generation and in resistance to fatigue.

**Figure 5:**
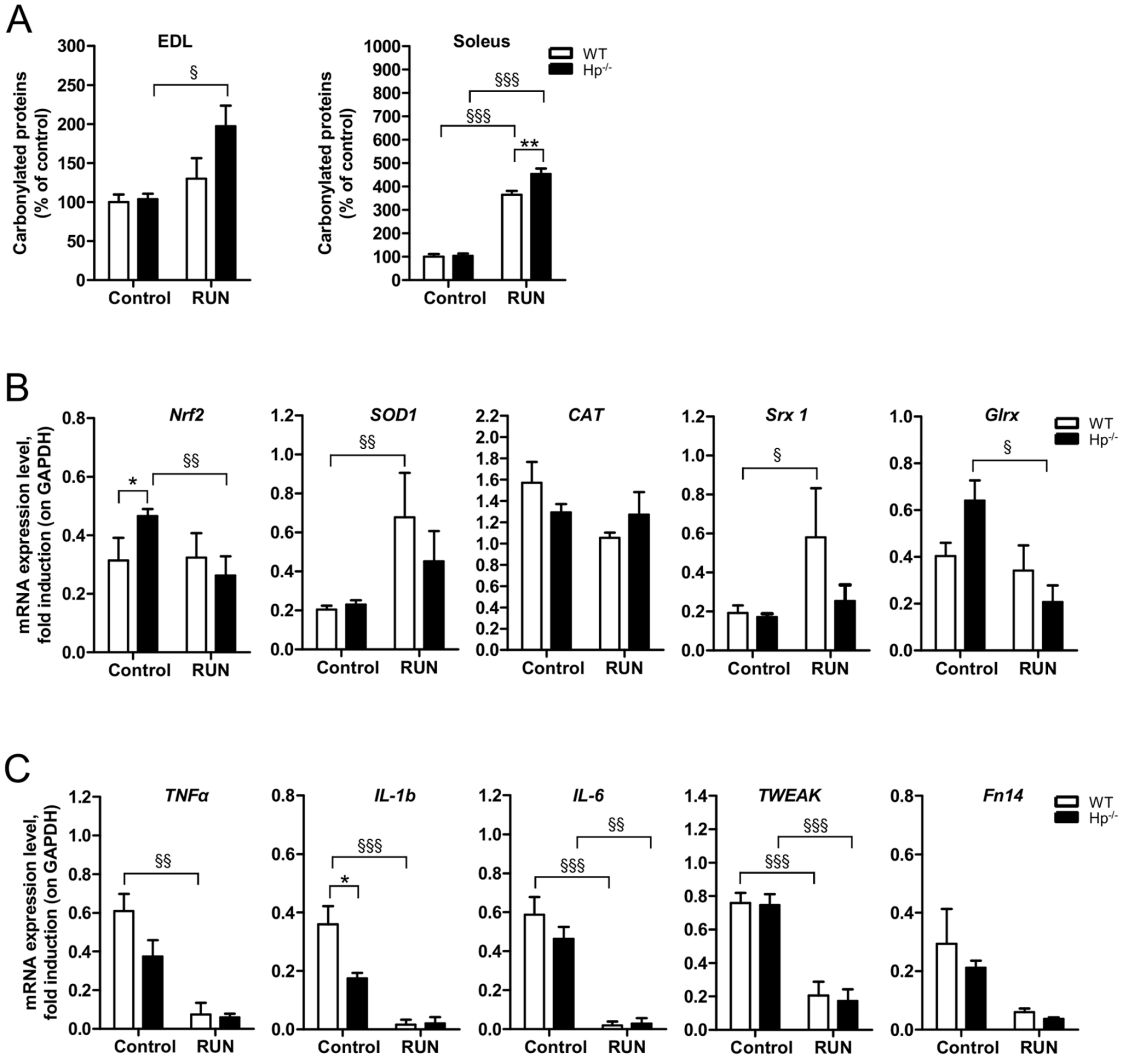
Following acute exercise Haptoglobin deficiency results in exacerbated oxidative stress and impaired antioxidant response. Each panel shows WT (white bars) and Hp^-/-^ (black bars) skeletal muscle in resting conditions (the same as figures 1 and 2, referred to as control, n = 6–8) and following 3 hours of rotarod exercise (referred to as “RUN”, n = 6). A) The exercise dependent increase in carbonylated proteins is more pronounced in the *EDL* and *Soleus* muscle of Hp^-/-^ as compared to WT mice. B) mRNA analysis for anti-oxidant response (names of genes as indicated) in *Tibialis anterior* muscle. C) mRNA analysis for inflammatory response (names of genes as indicated) in *Tibialis anterior* muscle. Data are expressed as mean ± SEM. *P<0.05; **P<0.01; §P<0.05; §§P<0.01; §§§P<0.001.

In order to understand the molecular mechanisms underlying Hp^-/-^ skeletal muscle weakness we monitored OS and signaling pathways. Exercise caused a significant enhancement of protein carbonylation in Hp^-/-^ mice both in *EDL* and in the *soleus* muscles (Figure 5A). In the latter the exercised dependent induction of OS was more evident and, to a minor extent, affected also the WT mouse. Interestingly, the boost of protein oxidation is associated with a reduction of *Nrf2* expression in Hp^-/-^ (Figure 5B), accompanied by a significant decrease of *Glrx* mRNA abundance. Conversely, exercised WT mice showed an activated anti-oxidant response with increased expression of *SOD1* and *Srx 1*. *CAT* transcripts were unchanged by exercise in both genotypes. Taken together these data indicate that the increased level of oxidized proteins seen in the skeletal muscle of exercised Hp deficient mice is mainly due on an inefficient anti-oxidant response.

Physical exercise has been previously associated to a diminished expression of inflammatory cytokines [33]. Indeed the effect of physical activity was remarkable in WT, which showed a significant reduction of *TNFα, IL-6, IL-1b* and *TWEAK*. The effect was present, albeit less pronounced, in Hp^-/-^ mice that showed a downregulation of IL-6 and TWEAK (Figure 5C).

**Figure 6:**
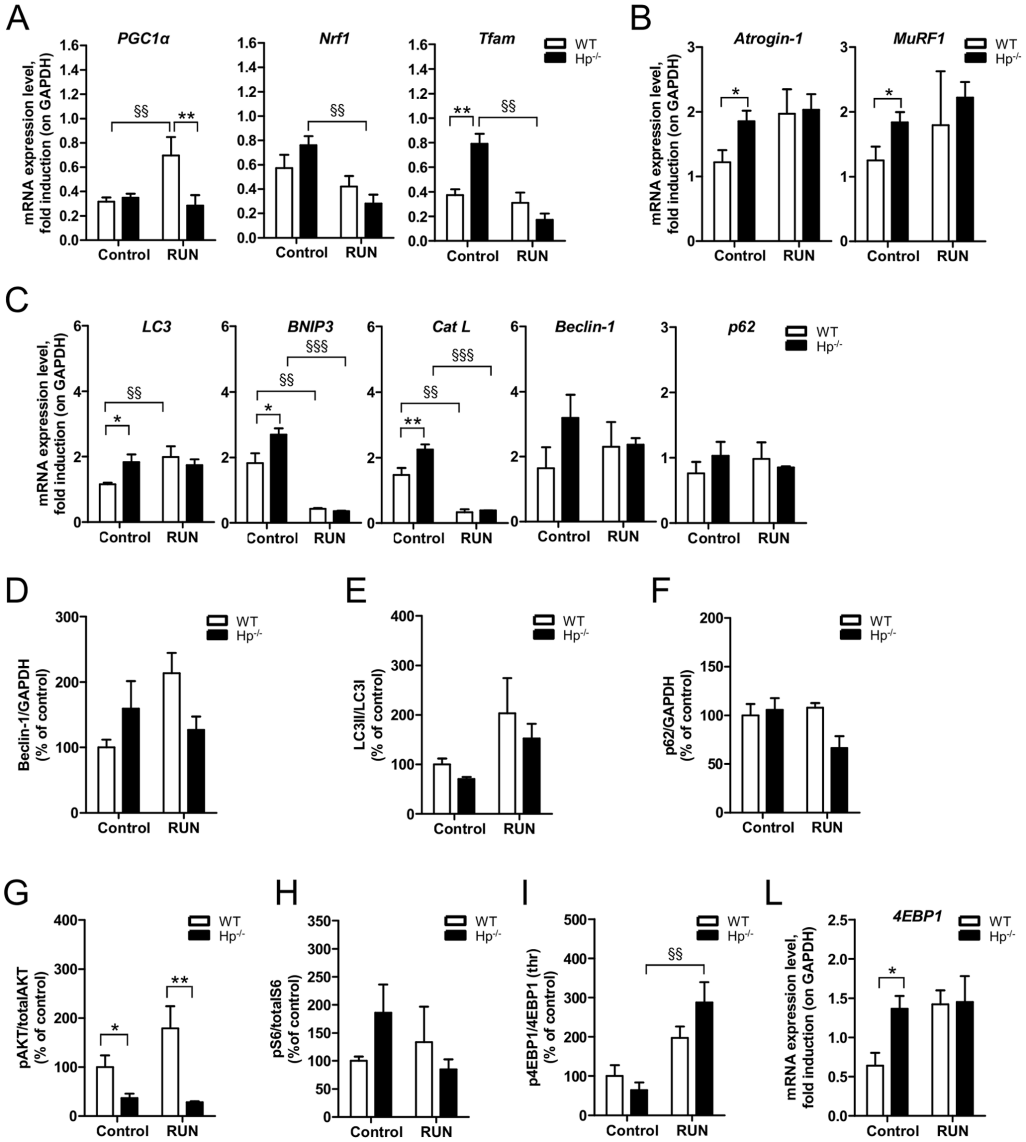
Following acute exercise Haptoglobin deficiency results in altered mitochondrial signaling in skeletal muscle. A) mRNA analysis for mitochondrial biogenesis (names of genes as indicated): *PGC1α* transcript is induced by exercise in WT mice and not in Hp^-/-^; *Tfam* and *Nrf1* are downregulated by exercise in Hp^-/-^ skeletal muscle. B) Exercise does not affect *Atrogin-1* and *MuRF1* mRNA levels in any of the two genotypes. C) mRNA amount of autophagy related genes (names as indicated) in control and RUN WT and Hp^-/-^ skeletal muscle. (D-I) Quantification of Western blots analysis on protein isolated from skeletal muscle of control and RUN WT and Hp^-/-^ mice; data are expressed as percentage of “control WT”, where control WT is considered 100%. D) Beclin-1 protein levels, E) LC3 lipidation (ANOVA treatment effect P<0.05), F) p62 protein levels, G) Akt phosphorylation (ratio between signal on Ser 473 and total Akt), H) S6 phosphorylation (ratio between signal on Ser 235/236 and total p70S6), I) 4EBP1 phosphorylation (ratio between signal on Thr 37/46 and total 4EBP1). J) *4EBP1* mRNA level. Real Time PCR and western blot analysis were respectively performed on *Tibialis anterior* cDNA and *Gastrocnemius* muscles protein lysates. GAPDH is used as control of equal loading in both cases. Muscles were dissected from 5 months old mice. Data are expressed as mean ± SEM. *P<0.05; **P<0.01; §§P<0.01; §§§P<0.001.

Exercise is a potent stimulus to promote mitochondrial biogenesis and to improve mitochondrial function and *PGC1α* is the master gene for mitochondrial function/biogenesis and for ROS detoxification. Moreover, *PGC1α* gene expression is activity dependent. When we monitored *PGC1α* expression we found, as expected, an induction in WT mice but not in Hp^-/-^ mice (Figure 6A). Exercise did not trigger changes of *Tfam* and *Nrf1* expression, two other factors involved in mitochondrial biogenesis, in WT mice. However, physical activity reduced *Tfam* and *Nrf1* levels in Hp^-/-^ mice. Altogether these findings suggest that exercise unmasked a pro-oxidative condition in Hp^-/-^, possibly coming from mitochondrial network, that determines weakness and fatigue.

Next we checked whether the pathways involved in protein degradation might play a role. Exercise did not significantly trigger the expression of *Atrogin-1* and *Murf1* in WT and Hp^-/-^ mice (Figure 6B). Since exercise on a treadmill is able to activate autophagy in adult muscles [34], we checked whether this happens also in our experimental model. Rotarod exercise was able to induce *LC3* expression (Figure 6C) in WT but not in Hp^-/-^ mice. Both genotypes showed a suppression of *Cat L* and *Bnip3* transcripts, while *Beclin-1* did not significantly change. However, Beclin-1 protein showed a trend of induction in WT mice (Figure 6D). When we monitored LC3 lipidation as readout of autophagy induction we found an exercise dependent induction (ANOVA, treatment effect P<0.05) in both genotypes (Figure 6E). When we looked at the anabolic pathway we found no further changes of Akt between sedentary and exercised animals. Hp^-/-^ mice showed a lower level of phospho-Akt than WT both in sedentary and exercised animals (Figure 6G). Exercise did not induce changes of p70S6k phosphorylation (Figure 6H) while it induced an increase of phospho-4EBP1 (Figure 6I) without changing the transcript expression of *4EBP1* (Figures 6L).

Taken together these data suggest that absence of Hp caused problem in mitochondria-related signaling pathways and increased OS, but did not trigger major alterations in signaling pathways related to protein breakdown and synthesis.

### Obesity induces muscle atrophy and weakness in Hp knockout mice via induction of autophagy-lysosome and ubiquitin-proteasome systems

We then studied the role of Hp as scavenger factor of OS when ROS are chronically higher, such as during systemic pro-inflammatory conditions like obesity. Mice were fed with HFD for 12 weeks. This resulted in elevation of plasma Hp by 3 fold in WT mice [10]. The two genotypes gained a similar amount of weight (89% and 82% of initial body weight for WT and Hp^-/-^ respectively). The final body weight was of 42.29±0.98 gr in HFD WT and 41.96±0.54 gr in HFD Hp^-/-^ [10].

**Figure 7:**
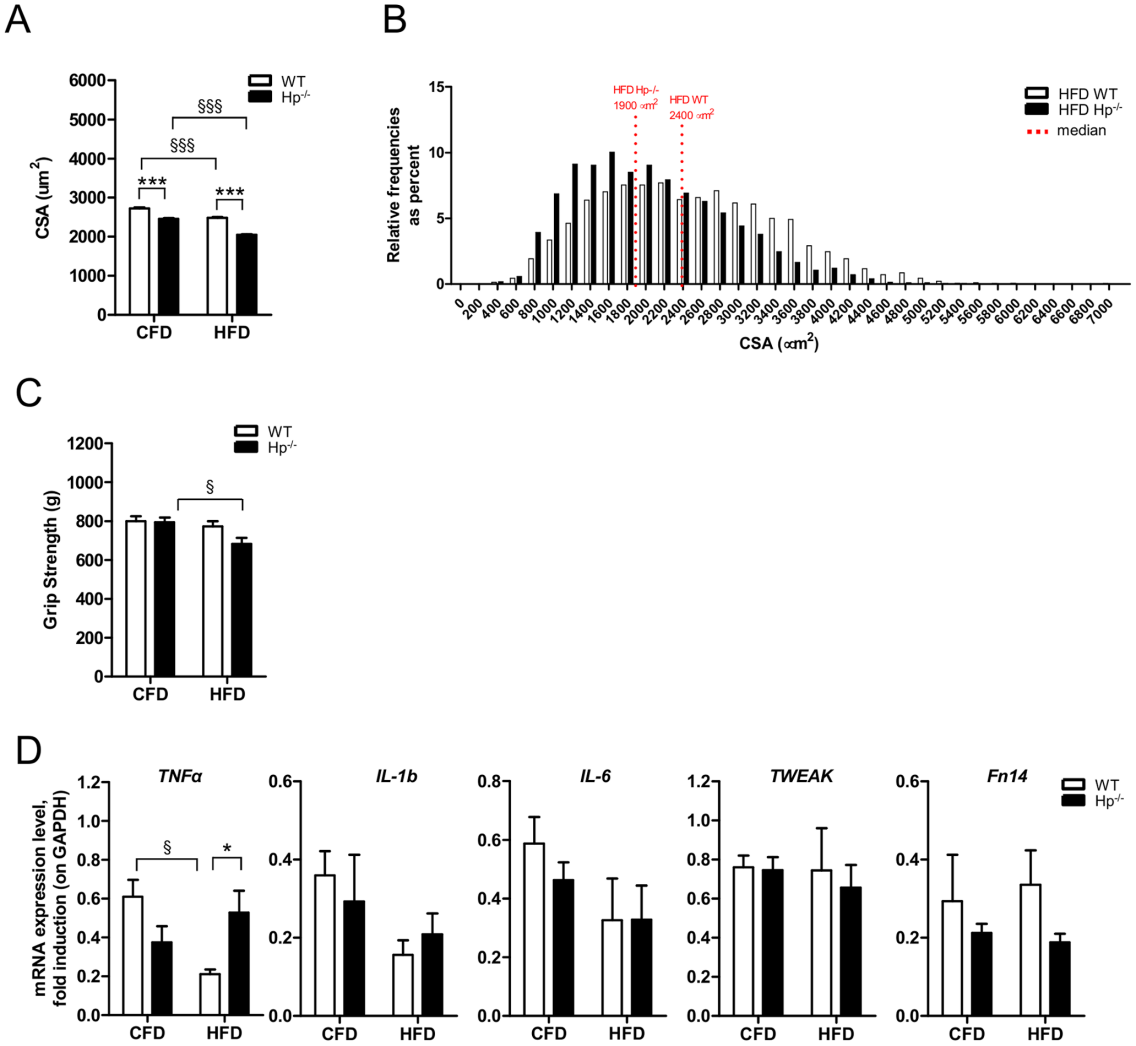
The effects of obesity on skeletal muscle size and performance in the absence of Haptoglobin. Each panel shows WT (white bars) and Hp^-/-^ (black bars) skeletal muscle under regular Chow Food Diet Feeding (the same as figures 1, 2 and 3, referred to as “CFD”, n = 6–8) and in the obesity condition following 12 weeks of High Fat Diet (referred to as “HFD”, n = 6). A) Hp deficiency exacerbates the obesity dependent reduction of *Tibialis anterior* cross sectional area (CSA) (n≥5000 fibers). B) Cross sectional area (CSA) frequency distribution of *Tibialis anterior* in obese Hp^-/-^ and WT mice. The bar graph indicates relative frequencies as percent. Red-dotted lines indicates the median for each distribution (n≥5000 fibers). C) Grip test assessment of forelimbs strength reveals an obesity dependent reduction only in Hp^-/-^ mice. D) mRNA analysis for inflammatory response (names of genes as indicated) in *Tibialis anterior* muscle. Data are expressed as mean ± SEM. *P<0.05; ***P<0.001, §P<0.05; §§§P<0.001.

It is well known that HFD triggers insulin resistance and muscle atrophy. While WT animals lost 10% of muscle mass following onset of obesity, the Hp^-/-^ mice doubled muscle loss compared to WT. Indeed Hp-deficient fibers were already 10% smaller and obesity caused a further 20% decrease: this effect can be clearly observed both in the comparison between the means (Figure 7A) and the distributions (Figure 7B). Consistently with morphology, obese Hp^-/-^ mice displayed a significant reduction of muscle strength as compared to CFD Hp^-/-^ (Figure 7C). Thus Hp deficiency upon HFD does not protect from muscle loss but exacerbates an already atrophic phenotype. Local inflammatory cytokines were unchanged by HFD in both genotypes except for *TNFα* that was significantly downregulated in WT mice (Figure 7D).

**Figure 8:**
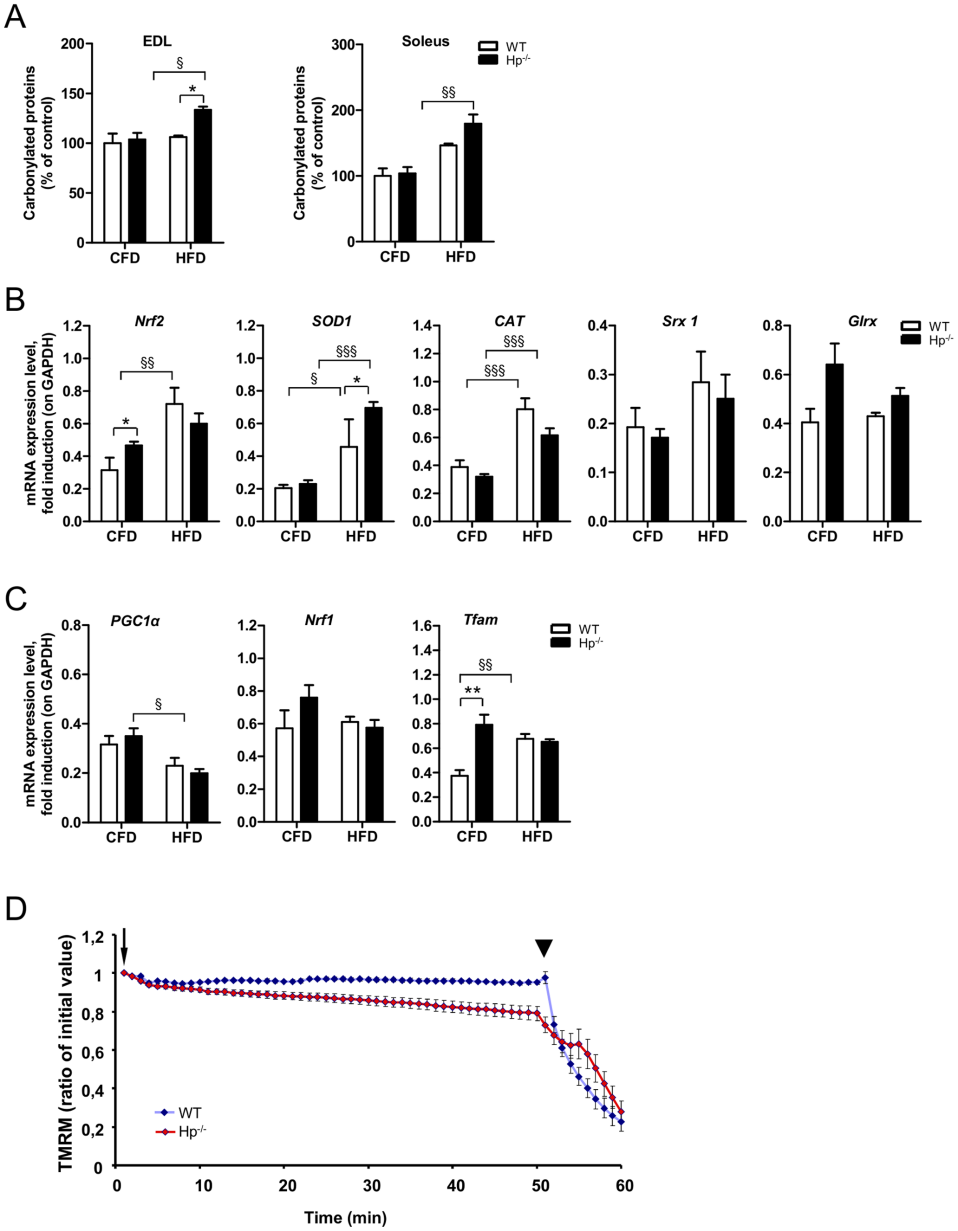
The effect of obesity on protein carbonylation, antioxidant response and mitochondrial function in the absence of Haptoglobin. Each panel shows WT (white bars) and Hp^-/-^ (black bars) skeletal muscle under regular Chow Food Diet Feeding (the same as figures 1, 2 and 3, referred to as “CFD”, n = 6–8) and in the obesity condition following 12 weeks of High Fat Diet (referred to as “HFD”, n = 6). A) Oxyblot reveals an obesity dependent increase in carbonylated proteins in the *EDL* and *Soleus* muscle of Hp^-/-^ mice. B) mRNA analysis for anti-oxidant response (names of genes as indicated) in *Tibialis anterior* muscle. C) mRNA analysis for mitochondrial biogenesis (names of genes as indicated) in the skeletal muscle: obesity dependent down regulation of *PGC1α* in HFD Hp^-/-^ mice, and *Tfam* upregulation in HFD WT animals. D) Mitochondrial response to oligomycin in single myofibers isolated from *flexor digitorum brevis* (FDB) skeletal muscles of WT and Hp^-/-^ mice. Where indicated, 6 µM oligomycin (arrow) or 4 µM of the protonophore carbonylcyanide-*p*-trifluoromethoxyphenyl hydrazone (FCCP) (arrowhead) were added. Traces represent tetramethylrhodamine methyl ester (TMRM) fluorescence as the mean of all the fibers of a group (n≥12). Fibers are considered as depolarized when they lose more than 10% of initial value of TMRM fluorescence after oligomycin addition. mRNA analysis and histological assessment are performed on *Tibialis anterior* skeletal muscle. Data are expressed as mean ± SEM. *P<0.05; **P<0.01; §P<0.05; §§P<0.01; §§§P<0.001.

HFD is known to induce systemic OS [35] and, directly or indirectly, to affect mitochondrial function [36]. When we checked the status of protein oxidation we found that carbonylated proteins were significantly increased in both *EDL* and *soleus* of HFD Hp^-/-^ mice but not of HFD WT mice (Figure 8A), even if there was a trend of induction in the *soleus*. Interestingly, *Nrf2* expression was significantly induced in obese WT animals but not in obese Hp^-/-^ animals with respect to relative controls (Figure 8B). The anti-oxidant genes *SOD1* and *CAT* were upregulated in both genotypes by HFD while *Srx 1* and *Glrx* did not significantly change. Interestingly, *PGC1α* was significantly downregulated in Hp^-/-^ mice by HFD (Figure 8C). The expression of other markers of mitochondrial biogenesis showed no major changes, except an upregulation of *Tfam* in WT obese animals (Figure 8C). Then we checked mitochondrial function by monitoring mitochondrial membrane potential in isolated single myofibers. Muscles of Hp^-/-^ mice showed a significant increased incidence of mitochondria that depolarized when the F_1_F_0_-ATPase was blocked by oligomycin [37] (Figure 8D). This finding means that mitochondria of animals lacking Hp are dysfunctional and have proton leak.

**Figure 9:**
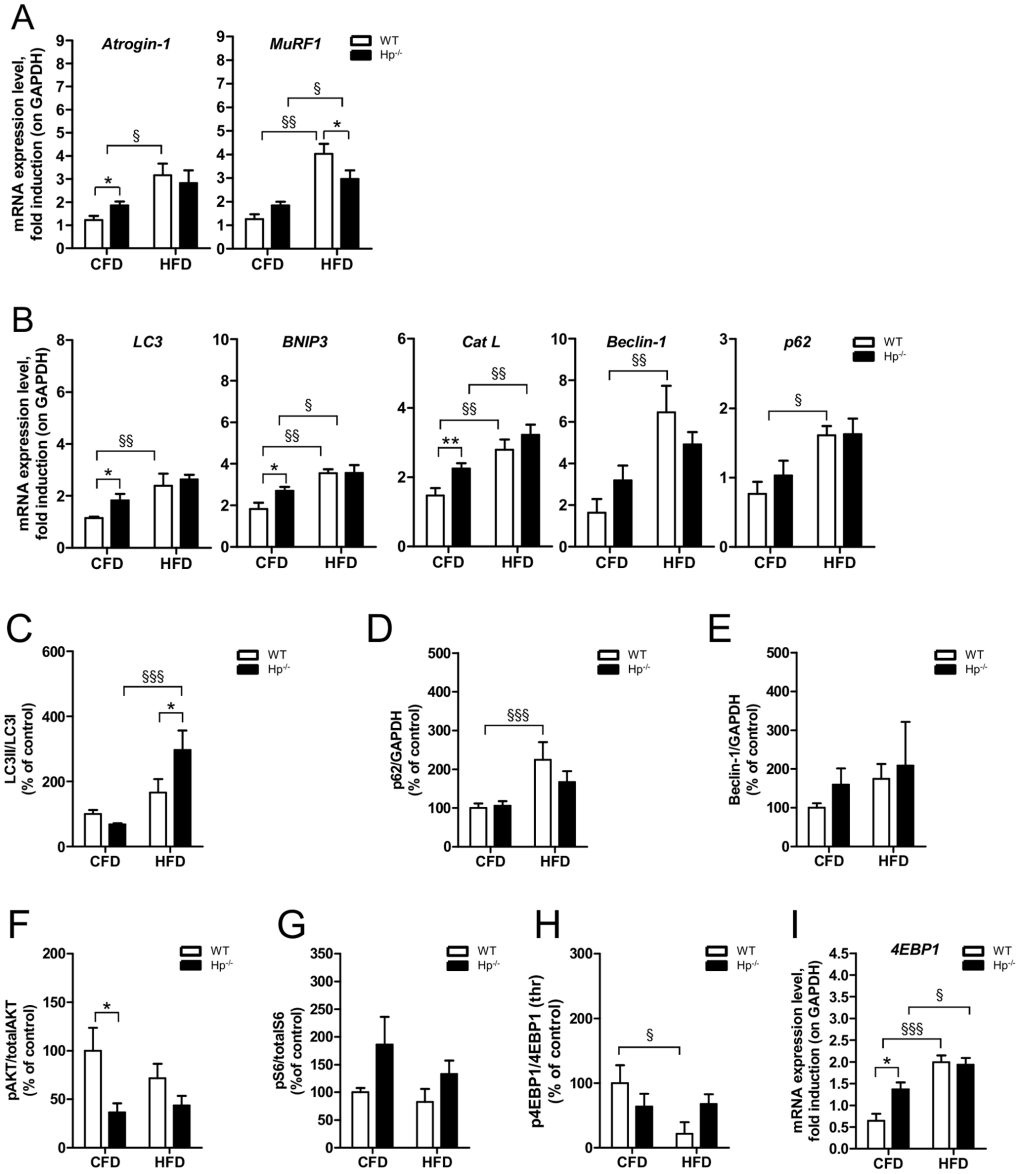
The effects of obesity on skeletal muscle protein balance in the absence of Haptoglobin. Each panel shows WT (white bars) and Hp^-/-^ (black bars) skeletal muscle under regular Chow Food Diet Feeding (the same as figures 1, 2 and 3, referred to as “CFD”, n = 6–8) and in the obesity condition following 12 weeks of High Fat Diet (referred to as “HFD”, n = 6). A) Obesity results in *Atrogin-1* transcript upregulation in HFD WT mice and in *MuRF1* transcript upregulation both in HFD WT and HFD Hp^-/-^ mice. B) mRNA levels of autophagy related genes (names as indicated) in CFD and HFD WT and Hp^-/-^ skeletal muscle. (C-H) Quantification of Western blot analysis on skeletal muscle of CFD and HFD WT and Hp^-/-^ mice; data are expressed as percentage of “CFD WT”, where CFD WT is considered 100%. C) LC3 lipidation, D) p62 protein levels, E) Beclin-1 protein levels, F) Akt phosphorylation (ratio between signal on Ser 473 and total AKT), G) S6 phosphorylation (ratio between signal on Ser 240/244 and total p70S6), H) 4EBP1 phosphorylation (ratio between signal on Thr 37/46 and total 4EBP1). I) *4EBP1* mRNA level is increased in both obese WT and HFD Hp^-/-^ skeletal muscle with respect to relative CFD. Real Time PCR and western blot analysis were respectively performed on *Tibialis anterior* cDNA and *Gastrocnemius* muscles protein lysates. GAPDH is used as control of equal loading in both cases. Muscles were dissected from 5 months old mice. Data are expressed as mean ± SEM. *P<0.05; **P<0.01; §P<0.05; §§P<0.01; §§§P<0.001.

We then monitored the ubiquitin-proteasome and the autophagy lysosome systems. HFD induced an upregulation of both *Atrogin-1* and *MuRF1* in WT mice, while only the latter was significantly induced in Hp^-/-^ (Figure 9A). Obesity caused an activation of all the autophagy-related genes in WT animals while only *Bnip3* and *Cat L* were induced in Hp^-/-^ mice (Figure 9B). Autophagosome formation, revealed by LC3 lipidation, was more robust in Hp^-/-^ than WT (Figure 9C). The level of p62 protein mirrored the mRNA trend (Figure 9D). Indeed, the accumulation of p62 protein in WT mice was not due to an autophagy-flux inhibition, but to a transcriptional induction of *p62* gene expression. No major differences were observed for Beclin-1 protein, even if there is a trend of induction in obese mice for both genotypes (Figure 9E). Altogether these findings suggest that obesity caused an activation of the ubiquitin proteasome pathway and of autophagy-lysosome systems in Hp^-/-^ mice, that account for their atrophic phenotype.

The anabolic Akt-mTOR signaling showed no changes in phosphorylation levels of Akt and S6 in both WT and Hp^-/-^ (Figures 9F, 9G). Obesity in WT induced a significant inhibition of 4EBP1 activity that may be caused by the important transcriptional upregulation of its gene (Figures 9H, 9I).

Altogether these findings indicate that the phenotype of obese Hp^-/-^ results from excessive protein breakdown rather than from alterations in anabolic pathways.

## Discussion

Conditions such as cancer, aging and obesity are characterized by chronic systemic inflammation and by muscle wasting. Sustained expression of inflammatory cytokines (TNFα, IL-6, IL-1b) is deleterious for muscle mass, because activates signaling pathways that promote protein breakdown and suppress protein synthesis, causing atrophy of muscle cells. During chronic inflammation two major mechanisms are believed to cause muscle atrophy, namely the increase of oxidative stress (OS) and insulin resistance. Both these conditions are described to activate an atrophy program and therefore muscle loss [38]. Excessive muscle loss is a highly detrimental state for the human body impairing therapies, aggravating diseases and increasing morbidity and mortality. Therefore, there is great interest in the scientific community in understanding the mechanisms that control muscle growth and muscle wasting. The inflammatory cytokines also evoke an acute inflammatory response consisting in the production and secretion of circulating factors, that are important and helpful for inflammation itself. However, some of these factors are also working as scavengers to prevent/limit some of the deleterious effects of chronic inflammation. How much important this systemic response is to reduce the side effect of the inflammatory cytokines on muscle mass is completely unknown.

Haptoglobin (Hp) is among the different factors that are secreted by the liver in response of TNFα and IL-6 stimulation [2], [39],[40]. Hp is an acute-phase plasma glycoprotein produced mainly produced by hepatocytes [1] and adipocytes [2], and is the major Haemoglobin binding serum protein [41]. An important aspect of Hp-Hb binding is to limit the pro-oxidative action of free Hb. The contribution of Hp to prevent OS and consequent muscle atrophy during chronic inflammation was unknown. We have explored this important action by characterizing adult skeletal muscles of Hp knockout (Hp^-/-^) mice. Moreover, Hp^-/-^ mice were also studied in conditions that are known to induce OS such as exercise and obesity. Interestingly, the signaling pathways activated by these two stresses are different.

In basal condition the Hp^-/-^ young mice do not exhibit any ouvert pathological feature, they are normally viable and fertile and their metabolic profile is not altered [10]. However, we found that maintenance of muscle mass is compromised at 5 but not at 3 months of age, suggesting an age-related phenotype. Therefore, Hp function seems to be dispensable in young age but starts to be crucial in adulthood. The decrease of myofiber size that occurs between 3 and 5 months of age is mainly due to upregulation of the ubiquitin proteasome system. The induction of the ubiquitin- proteasome system is consequent to AKT inhibition that leads to FoxO activation and transcription of the muscle-specific ubiquitin ligases Atrogin1 and MuRF1. Importantly, another FoxO target, *Bnip3*, is also significantly induced in knockout mice. Among the mTOR downstream targets only 4EBP is altered, suggesting that it might contribute to the phenotype of Hp^-/-^ mice through a slight reduction of protein synthesis. These findings well fit with the concept that muscle loss is mainly related to enhancement of protein breakdown instead of inhibition of protein synthesis [42], [43].

Notably the adult mice, in which the level of carbonylated proteins is not changed, show some signs of activation of an antioxidant response as revealed by a transcriptional upregulation of *Nrf2*. However, at this age the ROS scavenger genes that we analyzed were not significantly induced. We could only detect a trend of induction for *Glrx*. One potential interpretation is that in basal condition, at 5 months of age, the redox state starts to be moderately altered and activates the antioxidant response, which however is impaired or is just at the beginning of the induction. When animals were exposed to challenging, such as by exercise, the knockout became more prone to oxidative damage. Indeed both glycolytic and oxidative muscles showed an increase of carbonylation. This does not seem to be related to inflammation since exercise elicits an anti-inflammatory action in both genotypes. The susceptibility to oxidative stress in Hp deficient mice seems to be related to the inefficient activation of an anti-oxidant response. In fact, *SOD1* and *Srx 1* are induced in wild type but not in knockout while *Glrx* is suppressed in Hp^-/-^.

It is of interest to underline that *PGC1alpha* is not induced by exercise when Hp is absent. Therefore, it is possible that mitochondria of knockout mice start to generate more ROS due to the impaired PGC1alpha response and consequent alteration of mitochondrial fusion or biogenesis.

The signaling pathways related to degradation and anabolic signaling show minor changes that can not explain the exercise intolerance of Hp^-/-^. This finding implies that exercise affects mitochondrial network and ROS production, that need to be regulated in order to prevent alteration of sarcomeric proteins, that ultimately impact on force generation.

Obesity poses a different scenario from exercise. Similar to exercise, high fat diet leads to oxidative stress, but in this case *SOD 1* and *Catalase* are induced in Hp^-/-^mice. However mitochondria are greatly compromised in knockout mice and therefore, it is possible that the antioxidant response may not be sufficient to counteract mitochondrial-dependent ROS production. Differently from exercise, obesity induces autophagy and expression of *MuRF1*, suggesting that the degradation systems contribute to muscle atrophy in Hp KO mice.

The obesity-related activation of autophagy-lysosome system, together with the upregulation of *Bnip3*, an important gene for the selective removal of damaged mitochondria via autophagy (mitophagy) implies that this system may play an additional role in buffering mitochondrial dysfunction to limit ROS production and in inducing muscle atrophy [44]. Also in this case the changes of mTOR pathway are minimal and do not correlate with the phenotype of Hp-/-.

## Conclusions

These findings open a new view on the effects that pro-inflammatory cytokines elicit on muscle mass and ROS production. Here we provide evidence that inflammatory cytokines are also able to activate a response pathway (i.e. upregulation of Hp) that is important to reduce the detrimental effects of systemic inflammation and oxidative stress during catabolic or stress conditions.

## Methods

### Ethic statement

The experimental protocols followed the Principles of Laboratory Animal Care and a specific authorization was issued by Italian Ministry of Education (114/2003-A of 16/09/2003) to CNR Animal Facilities, where mice were housed. Mice were sacrificed by cervical dislocation following isoflurane anaesthesia (Abbott Laboratories, Kent, UK), and all efforts were made to minimize suffering.

### Experimental animals

The strategy used to target the Hp locus and generate Hp^-/-^ mice was described elsewhere [5]. Age matched littermates C57BL/6J WT were used as controls. When not otherwise specified 5 months old male mice were maintained on Chow Food Diet (these animals are referred throughout the text as CFD) (Diet 2018 Teklad global diet, 18.9% protein, 5.7% fat and 57.3% g/weight carbohydrate, Harlan, Indianapolis, IN). *Tibialis anterior*, *gastrocnemius* and *soleus* muscles were dissected from fed animals and frozen in liquid nitrogen for molecular assessment, and in 2-methylbutane for histological analysis. The number of animals studied in each experiment is indicated in the figure legends.

Animals referred to as *HFD* and *RUN* underwent treatments that are described below.

### High fat diet (HFD)

Animals were fed with HFD (19% protein, 36% fat and 35% carbohydrate g/weight, Diet F3282, Bio-Serve, Frenchtown, NJ) for 12 weeks. Animals had *ad libitum* access to food and water. Animals that did not reach 40 g of weight at the end of the diet, similarly represented in the two genotypes, were excluded from further investigations.

### Acute exercise, fatigue task [6]

Mice were trained for 2 days (day 1 and 2) on the rotarod RS (Harvard Apparatus, Holliston, MA) for acclimation purposes prior to the actual fatigue task. At day 1 mice were placed on the rotarod (speed 13 rpm) for 15 min. They were replaced on the rotarod if they fell off. After 30 min of rest, mice were placed again on the rotarod for 15 min with a speed from 13 to 20 rpm. At day 2 training regimen was similar to the day 1 protocol, but the speed was gradually adjusted from 13 to 20 rpm in a ramp of 15 min. The second run had a similar speed but the ramp was contracted to 180 s. At day 3 the fatigue task consisted of three rounds of 1 h each (20 rpm after a 180 s ramp, see also below) with a 15 min of rest between the first and the second task and 10 min of rest between the second and the third task. Mice were placed on the rotarod and ran at a speed ramping, in 180 s, from 13 to 20 rpm. They maintained a constant speed afterwards. The second task was similar to the first one. During the third task the speed was higher (13 to 21 rpm in a ramp of 180 s). If a mouse fell off four times within 60 s, the mouse was termed fatigued and the task was stopped. The number of falls and the duration time of consecutive exercising was recorded for each mouse. This experiment was performed in a blind fashion, with the operator unaware of the mouse genotype.

### Grip-strength test

Grip strength was measured each day at baseline and the end of the task. The grip-strength test apparatus consisted of a grasping trapeze connected to a force transducer (Ugo Basile, Varese, Italy). Each mouse was held by the base of the tail and placed in front of the grasping trapeze. Once the mouse grasped the trapeze, it was slowly pulled back until the pulling force overcame the mouse's grip strength. The grip-strength meter expresses the grip force in grams. Each mouse was submitted to five trials that were separated by an inter-trial interval of 15 min. Final score represents the average of the five trials for each mouse. The grip force was measured before and after the fatigue task both during the acclimation training and the actual fatigue task.

### Histological analyses

*Tibialis anterior* muscle was frozen in melting isopentane cooled in liquid nitrogen; 10 µm transverse sections were cut on a cryostat. Skeletal muscle frozen sections were subjected to Hematoxylin and Eosin (H&E) staining [45] or were stained for Succinate Dehydrogenase (SDH) activity using the succinic hydrogenase stain kit (cat. # 30-3014LY) from Bio-optica (Milan, Italy), following Manufacturer's instructions.

### Morphometric analysis

Fiber Cross-Sectional Areas (CSA) were measured using IMAGEJ software (Scion, Frederick, MD) by an observer unaware of the group assignments. A minimum of 1500 fibers evaluated in at least 3 different animals were analyzed for each group. The fields were randomly selected to measure the fiber area, and all the fibers encompassed in those fields were evaluated. This analysis was carried out in a blind fashion, with the operator unaware of the mice genotype.

### Immunoblotting

Muscles and cells were lysed and immunoblotted as described earlier [19]. Blots were stripped using, when needed, a stripping buffer (25 mM glycine, 1% SDS, pH 2.0) and re-probed. Antibodies are listed in table 1.

**Table 1:** Antibodies listed in alphabetical order.

Antibody	Customer	Dilution
rabbit anti-total Akt (# 9272)	Cell Signaling	1∶1000
rabbit anti-p-Akt (Ser473 #3787)	Cell Signaling	1∶500
rabbit anti-AMPK (# 2532)	Cell Signaling	1∶1000
rabbit anti-p-AMPK (Thr 172, # 2531s)	Cell Signaling	1∶1000
rabbit anti-Beclin-1 (# ab8245)	Santa Cruz	1∶1000
rabbit anti-GAPDH (#ab8245)	Abcam	1∶3000
mouse anti-LC3 (# 5F10)	Nanotools	1∶1000
guinea pig anti-p62 (# GP62)	Progen	1∶1000
rabbit anti-total S6 (# 2217)	Cell Signaling	1∶1000
rabbit anti-p-S6 (Thr389, # 2215s)	Cell Signaling	1∶1000
rabbit anti-4EBP1 (# 9452)	Cell Signaling	1∶1000
rabbit anti-p-4EBP1 (Ser65, # 9451)	Cell Signaling	1∶1000

### OXY blot

Protein lysates were prepared with the same procedure as for immunoblotting, but DTT was added to the lysis buffer to a final 50 mM concentration, as suggested in the manufacturer's protocol. OxyBlot Protein Oxidation Detection Kit (Millipore) was used for the procedure: 10 µg of protein were used per reaction.

### Single fibers mitochondrial membrane potential analyses

Muscle fibers were isolated from *flexor digitorum brevis* muscles. Mitochondrial membrane potential was measured by epifluorescence microscopy based on the accumulation of TMRM fluorescence, as previously described [9]. Fibers are considered as depolarized when they lose more than 10% of initial value of TMRM fluorescence after oligomycin addition.

### Real-time PCR (Gene expression analyses)

Total RNA was prepared from skeletal muscle using the Promega SV Total RNA Isolation kit. Complementary DNA generated from 400 ng of RNA with SuperScript III reverse transcriptase (Life Technologies), was analyzed by quantitative real-time RT-PCR using the QuantiTect SYBR Green PCR kit (Qiagen, Germany). Signals were normalized to GAPDH expression. Sequences of oligonucleotide primers used for SYBR green are shown in table 2. In the case of *Nrf2* (Mm00477784_m1), *SOD1* (Mm01344266_m1), *CAT* (Mm00437992_m1), *Srx 1* (Mm00769566_m1), *Glrx* (Mm00728386_s1), *TNFα* (Mm 00443258_m1), *IL-1b* (Mm 00434228_m1), *IL-6* (Mm 00446191_m1), *TWEAK* (Mm02583406_s1), *Fn14* (Mm01302476_g1), *PGC1α* (Mm01208835_m1), *Nrf1* (Mm00447996_m1) and *Tfam* (Mm00447485_m1) inventoried gene expression Taqman assays were purchased from Life Technologies-Applied Biosystems (CA, USA). A taqman assay for *GAPDH* (Mm99999915_g1) was employed for normalization.

**Table 2:** Primers used for SYBRgreen PCR.

Gene	Forward primer	Reverse Primer
*GAPDH*	ATACGGCTACAGCAACAGGG	TGTGAGGGAGATGCTCAGTG
*Atrogin-1*	TGGGTGTATCGGATGGAGAC	TCAGCCTCTGCATGATGTTC
*MuRF1*	CCTTCTCTCAAGTGCCAAG	CCTCAAGGCCTCTGCTATGT
*LC3*	CACTGCTGTCTTGTGTAGGTTG	TCGTTGTGCCTTTATTAGTGCATC
*Cathepsin L*	GTGGACTGTTCTCACGCTCAAG	TCCGTCCTTCGCTTCATAGG
*Beclin-1*	TGAATGAGGATGACAGTGAGCA	CACCTGGTTCTCCACACTCTTG
*Bnip3*	TTCCACTAGCACCTTCTGATGA	GAACACCGCATTTACAGAACAA
*p62*	CCCAGTGTCTTGGCATTCTT	AGGGAAAGCAGAGGAAGCTC
*4EBP1*	CCTCCTTGTGCCTCTGTCTA	GCCTAAGGAAAGATGGGTGT

### Statistical analysis

All values are expressed as mean ± Standard Error of the Mean (SEM). 2-tailed Student's t-test was used for pairwise comparisons. 1-way and 2-way or matched 2-way ANOVA followed by (when appropriate) Bonferroni *post-hoc* test were used to compare more than 2 groups. When only two groups were compared, the Student's t-test was applied assuming equal variances. Statistical evaluation was performed using GraphPad Prism 3 (GraphPad Software, USA). The difference among groups was considered significant at P<0.05.
